# Editorial for the Special Issue on Micro and Nano Devices for Cell Analysis

**DOI:** 10.3390/mi12070840

**Published:** 2021-07-19

**Authors:** Shohei Yamamura

**Affiliations:** Health and Medical Research Institute, National Institute of Advanced Industrial Science and Technology (AIST), 2217-14 Hayashi-cho, Takamatsu, Kagawa 761-0395, Japan; yamamura-s@aist.go.jp; Tel.: +81-87-869-3583; Fax: +81-87-869-4178

In recent years, miniaturized systems (micro- and nano-devices) called a lab-on-a-chip or micro-total analysis system (µ-TAS) have received attention as new systems for chemical and biochemical analyses. These devices are expected to perform DNA, protein, and cell analysis for drug screening and the development of novel diagnosis and therapies. In particular, micro- and nano-device technologies are expected to perform accurate and high-throughput analysis for the functions and characteristics of cells at single-cell or single-molecule levels. The developments of these micro- and nano-devices for cell analysis (cell chips) could lead to the next technology of drug screening and evaluation, disease diagnosis, and therapies. The history of cell chip devices began with cell and microorganism immobilization and patterning technology. Recently, cell separation, cell manipulation, cell culture, cell lysis, and gene amplification from cells (e.g., PCR) have been performed on various types of devices. In this Special Issue, we focus on novel devices or methodological developments of cell-based assay or cell-related (cell-derived) materials analyses. We are also interested in the technologies, materials, processes, and eventually applications of various assays (e.g., observation, manipulation, separation, detection, analysis) for cells (especially single cells).

This Special Issue of *Micromachines* entitled “Micro and Nano Devices for Cell Analysis” presents a total of 12 papers, including 10 articles and 2 reviews. Six papers are related to micro-device technologies (microfluidic, microarray, thin-bottom round-well plate, culture plate with a 3D lattice structure, micropipette, AFM with a micro-cup chip), and three papers deal with nano-device technologies (hole-arrayed plasmonic chip, LSPR sensor using nanopillars, plasmonic crystal (PC) with periodic nanostructures). One paper deals with unique chemical and biochemical materials for the control of cell manipulation. In 10 articles, 4 papers target single cells using their original device techniques. Two reviews focus on cell-based impedance spectroscopy (CBI) and electrochemiluminescence-based cell analysis systems, respectively.

In micro-device technologies, it is an advantage performing cell manipulation and analysis on a cell-size scale or single-cell level using various kinds of micro-devices. Yasukawa et al. [1] reported a new microfluidic device for the separation of red blood cells (RBCs) and white blood cells (WBCs) in a label-free manner based on negative dielectrophoresis (n-DEP). An alteration of the electric field generated by pairs of slanted electrodes is used to separate cells by their sizes. Using the microfluidic device, the separating efficiencies for RBCs and WBCs were found to be 91% and 93%, respectively. Shigeto and Yamamura et al. [2] developed a novel system to detect single-cancer cells expressing the T790M-mutated epidermal growth factor receptor (EGFR) mRNA from multiple non-mutated cancer cells by combining single-cell microarray chips and peptide nucleic acid (PNA)-DNA probes. Of the T790M-mutated cancer cells (NCI-H1975) that spiked into non-mutated cancer cells (A549), 0–20% were difficult for a conventional next-generation sequencer (NGS) and were quantitatively analyzed within 1 h. Therefore, this system could be useful in analyzing cancer tissue that contains a few anticancer drug-resistant cancer cells. Yamahira et al. [3] proposed a novel processing method in which an elastic mold made of polymethylsiloxane (PDMS) deforms to compensate for the dimensional error on the products. By heat-press molding a polycarbonate plate using a 384 PDMS convexes mold made from an acrylic sheet with 384 holes, a 384-round-well plate with a bottom thickness of 13.3 ± 2.3 mm (*n* = 384) was easily and accurately fabricated. Finally, single-cell polymerase chain reactions (PCRs) were demonstrated as the application of the products made by elastic PDMS molds. Ueno and Suzuki et al. [4] developed a cell culture plate with a 3D lattice structure, three micrometers in size, using a new thick negative photoresist SJI-001. SJI-001 showed the surface conditions for proper cell growth and low auto-fluorescence that does not affect fluorescence imaging. Therefore, SJI-001 exhibits surface conditions suitable for cell culture and is applicable for several applications for cell assay and analysis. Liu and Yue et al. [5] utilized a thin-neck-micropipette aspiration system to simultaneously quantify Young’s modulus and the specific membrane capacitance (SMC) value of six types of bladder cancer cell lines in different progression grades from the lowest metastatic potential G1 to the highest potential G4. The combination of these two physical properties on a scatter diagram clearly shows the cell groups with different cancer grades, which means that this combination could be a potential tumor grading marker to identify cancer cells with different metastatic potential. Kim et al. [6] measured changes in the mechanical properties of breast cancer cells (FP10SC2) before and after stimulation with macrophage-conditioned medium (MΦ-CM) in vitro. In addition, intercellular adhesion forces between the breast cancer cells were measured under the MΦ-CM stimulation using a micro-cup chip in atomic force microscopy (AFM). The results suggest that cancer cell malignancy was upregulated by tumor-associated macrophage (TAM)-like macrophage stimulation.

In nano-device technologies, it is an advantage performing high-sensitive analysis for cell-related or cell-derived materials (e.g., cell metabolites, cell membrane proteins) at a single-cell or single-molecule level. Yoshida and Tawa et al. [7] presented two types of membrane proteins (epithelial cell adhesion molecules (EpCAM) and epidermal growth factor receptor (EGFR)) in breast cancer cells (MDA-MB-231), which were observed using a hole-arrayed plasmonic chip with an epifluorescence microscope. As a result of multi-color imaging, the enhancement factor of fluorescence-labeled EGFR and EpCAM antibodies was over 13 and 12 times greater on the plasmonic chip than normal slide glass, respectively. Ali and Saito et al. [8] reported a simple microengraving cell monitoring method for neutrophil extracellular traps (NETs) released from single neutrophils. Such a method has been realized using a microwell array (MWA) sheet on a nanopillar-structured polymer-based localized surface plasmon resonance (LSPR) sensing chip. The Label-free LSPR sensing chips for the high-throughput detection of NETs released from single cells have been suggested to be useful in the study of NETosis for the detection and elucidation of autoimmune diseases. Endo et al. [9] proposed a surface-enhanced Raman scattering (SERS) active substrate as a plasmonic crystal (PC) with periodic noble metal nanostructures for the detection of phenobarbital, which is an antiepileptic drug. It shows that the evaluation of drug efficacy on human nerve cells or the body can be performed by the high-sensitive detection of phenobarbital using the PC device.

Furthermore, cell analysis devices combined with chemical and biochemical materials are also reported. Yamaguchi et al. [10] developed a photo-responsive surface for controlling the attachment and release of adherent cells on a substrate under light guidance. The surface comprises a poly (ethylene glycol) (PEG)-based photocleavable material that can conjugate with cell-adhesive peptides (arginine-glycine-aspartic acid (RGD) motif). The adhered cells were selectively released from the light-exposed region on the cell micropattern without damage. This study showed that the photo-responsive surface can serve as a facile platform for the remote-control of the patterning and recovery of adherent cells in micro-devices.

Two review papers introduced recent advances in cell analyses using various types of micro- and nano-devices combined with cell-based impedance spectroscopy (CBI) and electrochemiluminescence (ECL). Hassan and Kagan et al. [11] presented cell monitoring systems using cell-based impedance spectroscopy (CBI). This review gives a brief overview of the theory, instrumentation, and detection principles of CBI. The recent applications of the technique are given in detail for research into cancer, neurodegenerative diseases, toxicology as well as its application to 2D and 3D in vitro cell cultures. CBI is an established approach to non-destructively evaluate and perform the quality control of cell cultures with quantitative and sensitive data that can be easily adapted for single-cell analysis. This technique can be used in a variety of applications to analyze the effects of various therapeutic agents, nanoparticles and toxins for the pharmaceutical and environmental studies. Hiramoto, Ino and Shiki et al. [12] summarized recent advances in the electrochemiluminescence (ECL)-based systems developed for mammalian cell analysis. The review began with a summary of the developments in luminophores that related to ECL applications for biological samples. Secondly, ECL-based imaging systems were introduced as an emerging technique to visualize single-cell morphologies and intracellular molecules. The development of bipolar electrode (BPE) devices for ECL cell assays was also introduced. They concluded that the ECL technique is a highly versatile cell analytical platform in terms of diagnosis, being reusable, being low cost, and simplified devices exhibiting a high resolution and sensitivity.

Although some developed micro- and nano-devices are already commercially available, the sensing and diagnosis systems of biomolecules at the single-cell level remain a challenge. Therefore, the further development of these micro- and nano-devices for cell analysis in this Special Issue is expected to lead to the next technology of cell analytical tools, drug screening, diagnosis, and therapies.

We appreciate all the authors who submitted their precious and interesting research papers to this Special Issue. We would also like to acknowledge all reviewers for careful and timely reviews to ensure the quality of the Special Issue.
